# Processing and consolidation of open data on public procurement in France (2015–2023)

**DOI:** 10.1016/j.dib.2025.111277

**Published:** 2025-01-11

**Authors:** Adrien Deschamps, Lucas Potin

**Affiliations:** a Avignon Université, Laboratoire LBNC, 74 Rue Louis Pasteur, 84029 Avignon, France; b Avignon Université, Laboratoire Informatique d'Avignon, 339 Chemin des Meinajaries, 84000 Avignon, France

**Keywords:** Public procurement, Open data, E-procurement, Corruption, Green public procurement

## Abstract

Public procurement can be defined as the process by which public contracting authorities purchase goods, services, and works from private suppliers. To ensure transparency and prevent favoritism and corruption, public contracts adhere to strict procedures based on calls for tenders and award notices, which are publicly accessible online. This paper introduces a tabular dataset derived from the processing and consolidation of online public procurement notices. It provides a detailed overview of public contracts awarded in France between 2015 and 2023. The dataset encompasses approximately one million contractual relationships between public authorities and companies, in over 300,000 contracts, spanning all sectors and public institutions. It includes 113 variables covering contract characteristics (procedure, subject matter, award criteria, clauses, etc.), award outcomes (award price, number of bids, etc.), as well as information on contracting authorities (type, location, main activity, etc.) and awarded firms (size, legal status, main activity, age, location, etc.). This unprecedented dataset, both in accuracy and scope, provides reliable and detailed information on every advertised contract in France for nearly a decade, making it valuable for empirical research in diverse domains such as economics, geography, law, and political science.

Specifications Table

**Table:** 

Subject	Microeconomics
Specific subject area	The dataset provides comprehensive and accurate information on public contracts in France by processing raw data from online notices and consolidating it with individual data on public institutions and companies.
Type of data	Table Filtered, Processed, Enriched
Data collection	The data collection process begins by downloading notices from the website of the French official journal for public procurement. These notices are then centralized, and relevant information is extracted and processed. After that, missing identifiers of firms and contracting authorities are estimated, using a dedicated machine learning algorithm [1]. Finally, the data is consolidated by importing additional information on contracting authorities and companies, including foreign firms.
Data source location	The official website for public procurement notices (BOAMP): https://www.boamp.fr/pages/donnees-ouvertes-et-api/ Data on organizations and their location: https://www.data.gouv.fr/fr/datasets/base-sirene-des-entreprises-et-de-leurs-etablissements-siren-siret/ https://www.data.gouv.fr/fr/datasets/geolocalisation-des-etablissements-du-repertoire-sirene-pour-les-etudes-statistiques/ Data on municipal federations: https://www.insee.fr/fr/information/2510634
Data accessibility	Repository name: “BeauAMP: processing and consolidation of open data on public procurement in France (2015-2023)” Data identification number: 10.5281/zenodo.11001277 Direct URL to data: https://zenodo.org/records/11001277 Instructions for accessing the data: the dataset can be freely downloaded on the Zenodo repository. We recommend to open the Pickle file with Python. In addition, an equivalent CSV file is available, as well as CSV files for each year. The associated GitHub repository can be found at the following address: https://github.com/AdrienDeschampsAU/BeauAMP

## 1 Value of the Data

• The dataset enables the aggregation and analysis of data that was previously scattered across disparate documents. Since we directly use the information contained in online notices, our data features twice as many variables (113) as the similar database of Potin et al. (2023) [1], which was based on a partial selection of variables from the European Union's Tenders Electronic Daily (TED) tables. For example, the dataset may be used to chart the evolution of the implementation of environmental matters in public contracts between 2015 and 2023. In addition to descriptive statistics, the dataset may also be used to carry out econometric regressions with many control variables.
• The dataset addresses the inconsistencies of manually written notices and offers harmonized information across contracts. In particular, we use a list of keywords to categorize the award criteria (i.e., the different aspects of an offer evaluated by the contracting authority) and we standardize their weights (i.e., their relative importance in the score assigned to each offer) on a scale from 0 to 100% of the global score.
• The dataset provides detailed descriptions of the organizations, both public and private, involved in public procurement (staff size, legal status, creation date, main activity, etc.). For instance, one may want to study the contracts awarded to SMEs, or only municipal procurement, which requires to identify SMEs among the awarded firms and municipalities among contracting authorities.
• Our dataset includes the GPS coordinates of public and private organizations involved in public procurement, including foreign firms. The data can be used to generate maps or conduct spatial analysis. For instance, the data could be utilized to measure the average distance between contracting authorities and their private suppliers, or to map international exchanges in public procurement.
• The dataset can be linked to countless further sources of information. Indeed, the official SIRET (*Système d'identification du répertoire des établissements*) and SIREN (*Système d'identification du répertoire des entreprises*) identifiers of contracting authorities and firms serve as widely used keys for identifying entities across various databases. The information contained in our dataset opens up opportunities for research in diverse fields, as it may be connected to the official trade register, the European Union Emissions Trading System, data on gender equality in companies, and more.
• We chose a tabular format to make the data handling as easy as possible, including for non-expert users. Additionally, the related Python code is accessible and may be freely modified to enhance, update, or adapt the dataset for future research: https://github.com/AdrienDeschampsAU/BeauAMP.

## 2 Background

This dataset has been developed in response to an increasing need for empirical analysis of public procurement. On the one hand, the OECD estimates public procurement to represent one third of public expenditure in the world [2]. It involves national governments, local governments, and diverse public agencies. On the other hand, public procurement is becoming a major public policy instrument, transcending its traditional role as a supply lever. In many countries, including France, governments expect contracting authorities to pursue new goals, such as sustainable development, innovation, or the promotion of SMEs. Both the financial amount and growing complexity of public procurement make empirical investigation necessary to guide the utilization of public funds. The size and diversity of our dataset may lead to many research of general interest: SME access, sustainable public procurement, favoritism and corruption prevention, etc.

Researchers have already attempted to build datasets based on national [1,3] or international [4,5] open data on public procurement. However, these works usually rest on partial sources [1,4] or focus on specific sectors [3]. The purpose of our work is to provide an unprecedentedly large and accurate dataset to promote the empirical analysis of public procurement in France, by using the source of information closest to the contracting authority. In particular, we would like to update and improve the similar work of Potin et al. [1] in France (2010–2020), by directly using public procurement notices.

## 3 Data Description

### 3.1 Structure of the dataset

**Fig. 1:**
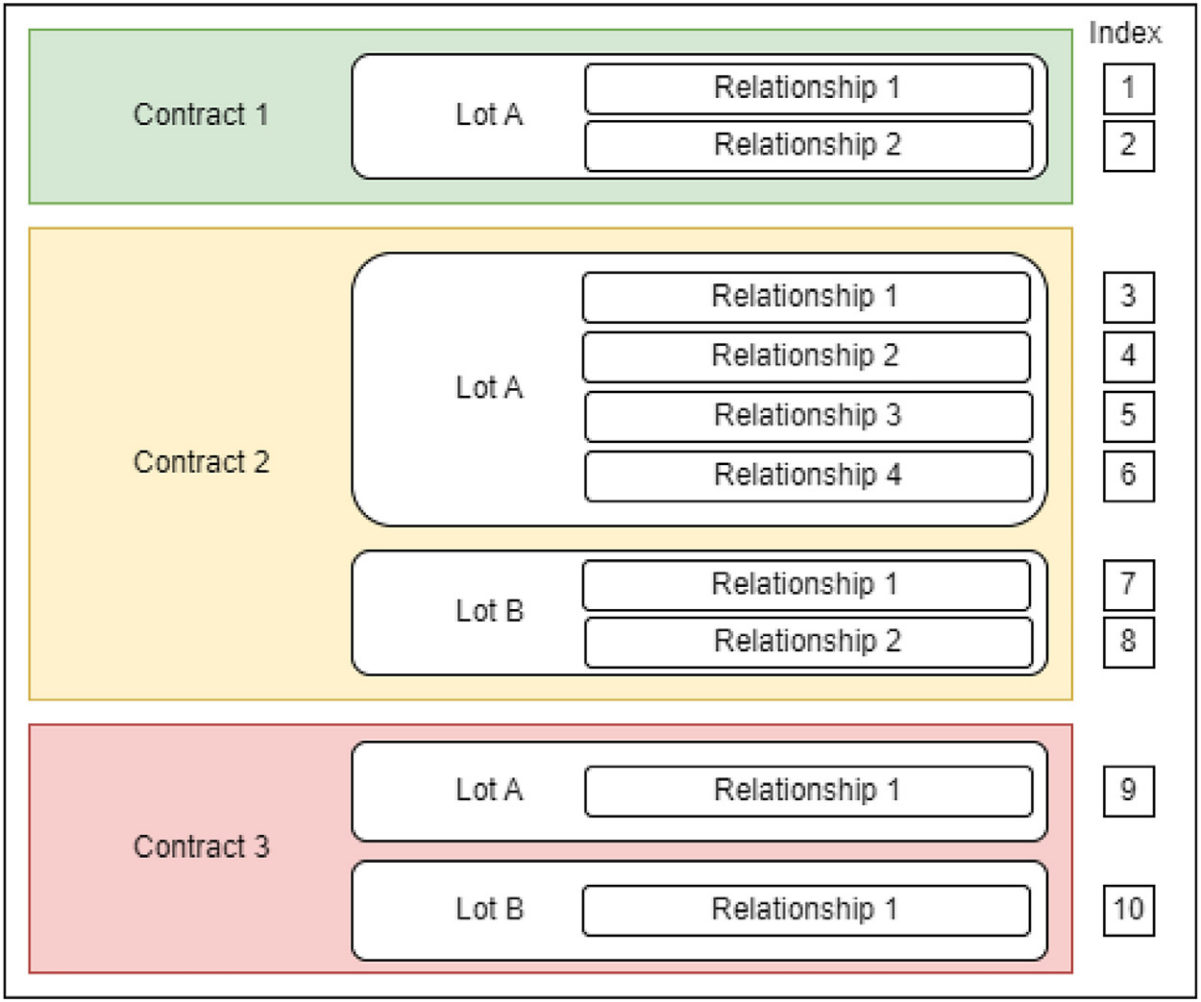
structure of the dataset.

The dataset is in a tabular format. It consists of 1,162,969 rows and 113 columns. Each column corresponds to a variable, as described in the following subsection. Each row corresponds to a contractual relationship between a contracting authority and a firm. Fig. 1 summarizes the structure of the data. A lot corresponds to a contract subdivision. Public contracts are typically divided into multiple lots to encourage competition and SME access to public procurement. In the example, 'Contract 2′ and 'Contract 3′ are divided into two lots, while 'Contract 1′ is not. Additionally, it is important to note that one lot may be scattered across multiple rows in the dataset, if several companies were awarded the same lot (either as part of a business association or as independent entities). For example, there are two observations for the single lot 'Contract 1′, as two contractual relationships were established for this lot. Conversely, each lot in 'Contract 3′ was awarded to a single company, resulting in only one row in the dataset for each lot.

### 3.2 Variables

The dataset comprises 113 variables, which can be divided into four categories: information on the contract, information on the outcome of the award procedure, information on Contracting Authorities or Entities (CAEs), and information on awarded companies. Due to the large number of variables, they will be presented in detail in the 'variable_description' file for ease of reading. It provides the name, type, content and availability of each variable in the dataset.

## 4 Experimental Design, Materials and Methods

**Fig. 2:**
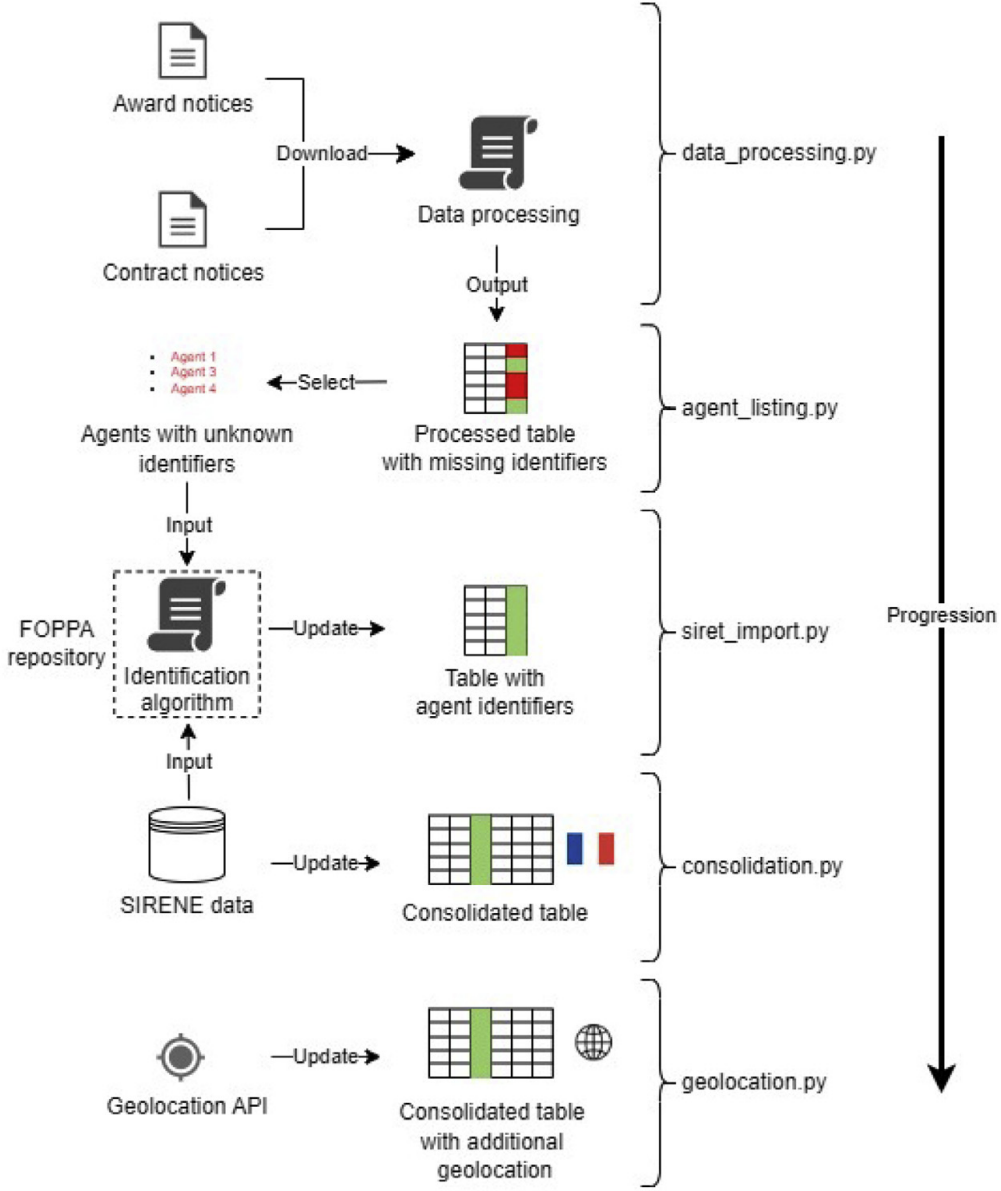
summary of the process.

This section provides an overview of the data collection, processing, and consolidation. As highlighted by Fig. 2, we begin by downloading contract and award notices from an online official website. We then process the extracted data into a tabular dataset. After that, we estimate the official identifiers of contracting authorities and firms when they are missing, so that we can consolidate our data with the features of French organizations as they appear on the official agent database. Finally, we attempt to track the GPS locations of the foreign firms mentioned in the dataset.

### 4.1 Contract and award notices

In France, the BOAMP (*Bulletin Officiel des Annonces des Marchés Publics*) is the official journal where contracting authorities publish notices of their procurement contracts when their value exceeds the legal thresholds. The purpose of advertising is to prevent favoritism and ensure the accountability of contracting authorities in the use of public funds. Contracting authorities typically begin by publishing contract notices (call for tenders) to inform potential bidders about the upcoming contract and award process. The outcome of the procedure is then announced in an award notice. Our dataset was generated using these two types of notices downloaded from the BOAMP website, between January 1, 2015, and December 31, 2023.

### 4.2 Data processing

We download contract and award notices as JSON files from the BOAMP website. Relevant information is directly extracted from the notices. In addition, we process textual data to improve the handling of award criteria. Like Potin et al. (2023) [1], we had to face the heterogeneous descriptions of award criteria among the award notices. Indeed, contracting authorities are not required to follow any standard format for this information, even though it is a decisive aspect of awarding procedures. To address this issue, on the one hand, we standardize the weights of the award criteria. On the other hand, we suggest a categorization of qualitative award criteria (as opposed to the price criterion) based on a list of keywords.

### 4.3 Missing individual identifiers

In France, both public authorities and companies are assigned national identifiers, namely the SIRET and the SIREN. The SIREN corresponds to the first nine digits of the 14-digit SIRET. While the SIREN corresponds to the company or public institution as a whole, the SIRET refers to a specific establishment of the agent, thus introducing a geographical dimension. Ideally, the contract notices should mention the identifiers of contracting authorities and companies. However, only 25 % of the notices provide the SIRET of the contracting authority, and 6% indicate the SIRET of the awarded firms. To estimate these missing SIRETs, we will use the available information on contracting authorities and firms (stated name, address, city, and postal code) as input for the aforementioned machine learning algorithm [1].

### 4.4 Data consolidation

After estimating the identifiers of contracting authorities and firms, we consolidate our dataset with individual characteristics on organizations based on their SIREN and SIRET, such as creation date, legal status, staff size, GPS position, and more. This information comes from the national SIRENE (*Système national d'identification et du répertoire des entreprises et de leurs établissements*) database, which provides detailed information on national economic agents.

### 4.5 Locating foreign firms

The final step consists in trying to locate foreign companies, using their address or country code, as foreign institutions are not included in the SIRENE database. We use a two-step process to estimate their GPS coordinates. First, we input the companyʼs stated address into the "Nominatim" API. If this initial attempt fails, we look for the average GPS coordinates of the corresponding country based on the ISO country code provided in the award notice.

## Limitations

The notices published by the contracting authorities are the original source of information for our dataset. As a result, the data is subject to the imperfections and errors present in the online notices. As highlighted by Tables 1 to 4, some variables are often omitted by the civil servants responsible for writing the notices (e.g., the minimum and maximum values of tenders). While we do not attempt to correct the work of civil servants, our dataset accurately reflects the information mentioned in the original notices. This claim can be easily verified by manually comparing our dataset with the online data for a random contract. Moreover, the quality of the original notices has improved over time.

Although the identification machine learning algorithm is a very useful tool for consolidating public procurement open data, it is not perfectly reliable. Potin et al. [1] highlight that it accurately identifies approximately 80% of the SIRETs. While the algorithm reduces the margin of error by searching within specific areas and treating contracting authorities and firms separately, it is important not to blindly trust the estimated identities if they seem surprising. We believe our dataset is better suited for conducting large-scale assessments than for focusing on individual cases. However, many key variables in the dataset are independent of the agent identifiers (especially the variables mentioned in Tables 1 and 2), so that their reliability is not affected by the efficiency of the machine learning algorithm.

Finally, the dataset focuses only on one country, unlike other comparable works [4,5], and it would be very valuable to have similar works for more regions.

## Ethics Statement

The authors have read and follow the ethical requirements for publication in Data in Brief and confirms that the current work does not involve human subjects, animal experiments, or any data collected from social media platforms.

## CRediT authorship contribution statement

**Adrien Deschamps:** Conceptualization, Methodology, Software, Writing – review & editing, Writing – review & editing. **Lucas Potin:** Resources.

## Data Availability

ZenodoBeauAMP : processing and consolidation of open data on public procurement in France (2015-2023) (Original data).

## Appendix Supplementary materials

Image, application 1
